# When discussing the impact of AI on radiology, just remember: radiology is an infinite game

**DOI:** 10.1590/0100-3984.2020.53.6e2

**Published:** 2020

**Authors:** Felipe Soares Torres

**Affiliations:** 1 MD, PhD, Department of Medical Imaging, University of Toronto, Toronto, ON, Canada. Email: felipe.torres@uhn.ca.

Over the past few years, headlines suggesting the demise of radiology under the rise of artificial intelligence (AI) have spread in specialized and lay media. One of the most emblematic testimonials came from Professor Geoffrey Hinton of the University of Toronto, an iconic researcher on neural networks and machine learning. In 2016, professor Hinton stated that “people should stop training radiologists now” and “it is just completely obvious that within 5 years deep learning will do better than radiologists”^(1)^. The impact of the bad news has been noticed by medical students. A considerable proportion of medical students have been discouraged from considering radiology solely because of the uncertain impact of AI on the field^(2,3)^. Indeed, the notion that AI may negatively impact radiology in the near future permeates the medical imaging community^(4)^. When such discussion emerges, many of us forget one of the most important factors when analyzing the potential demise of radiology in the face of AI: radiology is an infinite game.

The concept of finite and infinite games was introduced in the 1980s by author James P. Carse^(5)^. A finite game, among other characteristics, has known players, known rules (rules to which players have agreed upon) and a clear definition of when the game ends. Players play to win. Many sports, including football and hockey, are finite games. There is a beginning, a middle, and an end, with a winner and a loser. In contrast, infinite games are those with known and unknown players, no exact set of rules, in which a player leaves the game only when low on resources or no longer willing to play. Players play to keep playing (i.e., to perpetuate the game). There is no finish line, no winner, and no loser. This concept has been extensively analyzed by Simon Sinek in the business world^(6)^, with many examples of the application of this concept in the real world. It is no different in radiology. We most often do not know all the players (radiology groups, radiology practices, non-radiology physicians, hospitals, etc.). The players determine their own strategies, and, other than ethical, moral, and legal guidelines, which may vary from country to country, there are no fixed rules to which everyone has agreed. We never win or lose. In addition, we never see a finish line. Even if there is a finish line for an individual patient or for our daily tasks, the game lives on. Therefore, the game of radiology fits the definition of an infinite game.

Radiology is a field of medicine that deals with imaging. The definition of radiology may vary depending on the source. For instance, the Merriam-Webster dictionary defines radiology as “a branch of medicine concerned with the use of radiant energy (such as X-rays) or radioactive material in the diagnosis and treatment of disease”^(7)^. This apparently outdated definition makes evident the transformation radiology has gone through since the discovery of X-rays in the nineteenth century. Radiology has evolved to incorporate multiple imaging modalities and to become a medical imaging specialty. Indeed, the website radiologyinfo.org, the public information website developed and funded by the Radiological Society of North America and the American College of Radiology, defines a radiologist as a physician who completed medical school and received specialized training in obtaining and interpreting medical images obtained through the use of X-rays (radiographs, computed tomography, fluoroscopy, etc.), radioactive substances (nuclear medicine), sound waves (ultrasound), or magnets (magnetic resonance imaging)^(8)^. However, if we stick with the definition of what radiology means to understand what radiologists do, we will perhaps miss a crucial point: the overarching purpose of radiology. As a medical specialty, we do what we do to achieve a higher goal, to advance a cause. The cause is always centered on the patient. In medicine, as in radiology, our most important focus is the patient.

Given the fact that the cause radiology is always advancing is centered on the patient, discussing the potential impact of AI raises one question: why would AI or any other innovation be a barrier for radiology to advance its cause? This question only makes sense because of the challenges radiologists are facing on a daily basis. Pressure from many sides, including reimbursement models, organizational models of radiology practice groups, and the role played by radiological societies, may interfere with the pursuit of advancing the cause of radiology. AI would then be just another side. However, radiologists should look at this high-pressure environment as fertile ground to promote and advance their cause. For instance, radiologists should embrace value-based care as opposed to a fee-for-service model, because the former is more clearly linked to the advancement of patient-centered care. Group-based practices must define clear metrics to evaluate their services and initiatives in light of the overarching goal of promoting patient health. Any initiative, such as increasing contact with patients or with referring teams, should be linked to a measurable, patient-centered outcome. Radiological societies may fine-tune their roles, devoting more resources to pursuing the goal of advancing patient care.

As an infinite game, radiology has no winners or losers. We are not competing with anyone or anything. We are always looking to use the best evidence, judgment, and imaging modality to optimize the benefits to the patient. In this scenario, AI cannot and will never cause the demise of radiology. AI can only be seen as a tool that may help us advance our cause. If it is proven to be helpful, we will use it because it will make us better at what we do. It if proves superfluous or redundant, it will not be embraced by the medical imaging community. Notably, a finite mindset, in which a win-lose scenario is in place, cannot define the role of radiologists in medicine. Finite games may indeed exist in the infinite game journey, but they are secondary. However, the use of a finite mindset to try to understand the future of medicine or radiology, particularly in the context of a disruptive innovation, is doomed to fail, sooner or later. In fact, as pointed out by Simon Sinek, “disruption is a symptom of a finite mindset”^(6)^. That is true even if it is seen in retrospect.

If the definition of radiology changes, as it has since Wilhelm C. Roentgen discovered the X-ray more than a century ago, the purpose of radiology has never moved an inch from its conception. That is why we keep playing, even in periods of apparent uncertainty, unfaltering in our willingness to play. Therefore, AI and many other technological innovations that have emerged along the course of our specialty will never be able to supercede or cause the demise of radiology. The reason is simple: radiology is an infinite game.
